# Supplementary material for: Ultrafast generation and decay of a surface metal
Source: Nat Commun. 2021 Feb 12;12:978. doi: 10.1038/s41467-021-21203-6 (PMC7881126; doi:10.1038/s41467-021-21203-6)
Supplement: Supplementary file 1 — Supplementary Information [file 41467_2021_21203_MOESM1_ESM.pdf]

Supplementary Figures for

Ultrafast generation and decay of a surface metal

L. Gierster[1,2*], S. Vempati[1,3], and J. Stähler[1,2]

[1]Fritz-Haber-Institut der Max-Planck-Gesellschaft, Abt. Physikalische Chemie, Faradayweg 4-6, 14195 Berlin, Germany
[2]Humbolt-Universität zu Berlin, Institut für Chemie, Brook-Taylor-Str. 2, 12489 Berlin, Germany
[3]Present address: Department of Physics, Indian Institute of Technology Bhilai, Raipur-492015, India
*corresponding author

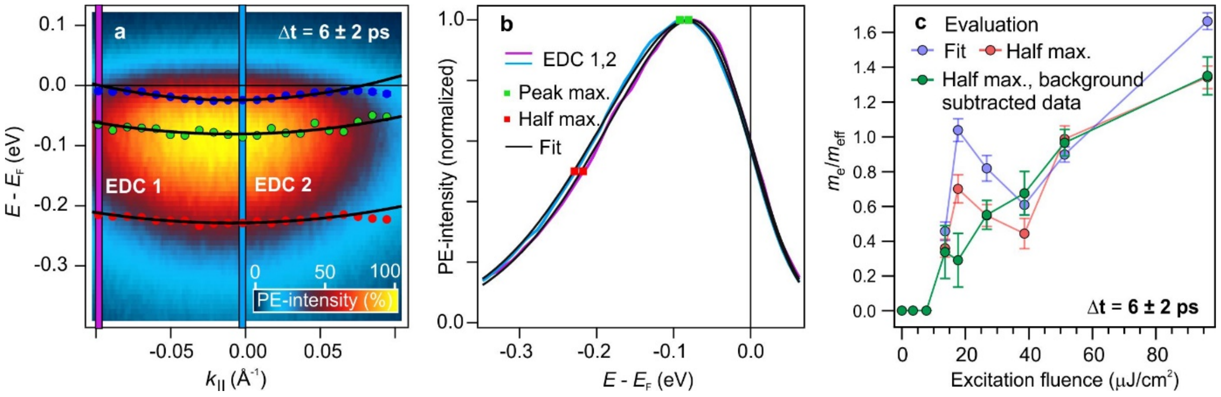

Supplementary Figure 1 Different evaluations of the angular distribution of the photoresponse. a, Angular distribution at a delay of 6 ± 2 ps after resonant photoexcitation with a fluence of 27 µJ/cm^2. Blue: Peak positions from fitting energy distribution curves at different $k_{\parallel}$ (e.g. EDC 1, EDC 2 as indicated with the blue/purple boxes) with a Gaussian peak multiplied by a Fermi-Dirac distribution and convolved with another Gaussian peak to account for the energy resolution (50 meV). Fits to exemplary spectra are shown in **b**. Green: Peak maximum of the EDC, red: position of the low energy edge half maximum, numerically determined, as shown in **b**. The black lines in **a** are fits with $E(k_{\parallel})=E_0 + \hbar^2 k_{\parallel}^2/(2m_{\mathrm{eff}})$; m_{eff} = 1.2(1) m_e (blue), m_{eff} = 1.7(5) m_e (green) and m_{eff} = 1.8(3) m_e (red). **c**, Comparison of the curvature for different fluences; error bars represent standard deviations. Blue: Evaluation from fitting. Green and red: evaluation by determining the half maximum numerically. For the green data points the background at negative delays was subtracted. For low fluences smaller than the PIPT threshold, the background has to be subtracted in order to identify the (localized) character of the excitation, because the photoinduced change is small. Clearly, independent of the evaluation method, we find a free-electron-like band with positive curvature and that the curvature increases as a function of photodoping.

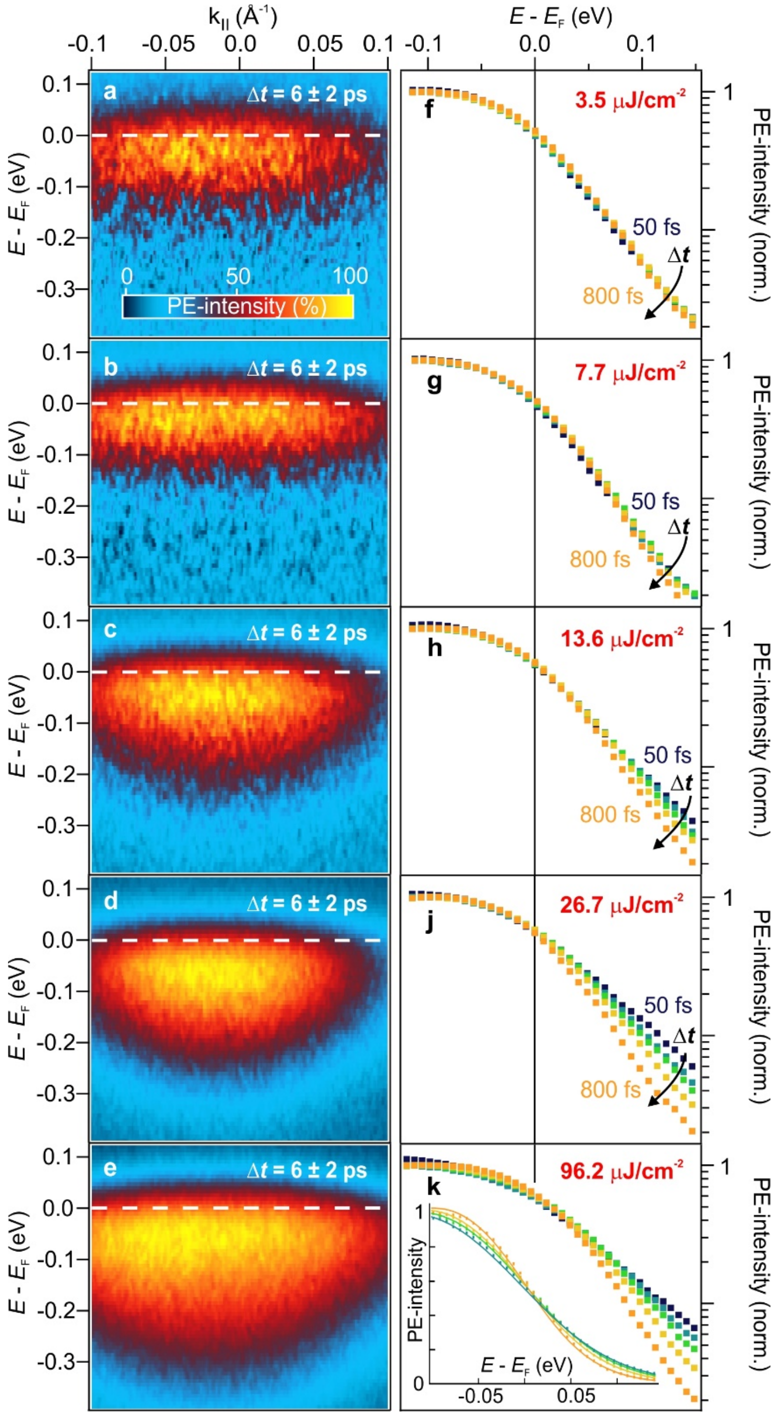

Supplementary Figure 2 Fluence dependence of the photoinduced response. a-e, Angular distribution of the pump-induced change at 6±2 ps below and above the PIPT threshold F_C (13.6 µJ/cm^2). The photostationary signal is subtracted to show the low fluence response. Below F_C, the pump laser pulse induces a non-dispersive state below E_F. Above F_C, a dispersive band evolves, with increasing bandwidth and curvature. For all fluences, the band is cut by the equilibrium Fermi level. **f-k**, Corresponding angle-integrated PE-spectra (log. scale, normalized) for different pump-probe delays (50 fs to 800 fs). Below F_C, all spectra are identical. Above F_C the high energy tail shows cooling of a hot electron population by equilibration with the lattice, characteristic for photoexcited metals. The delay dependence is described by Fermi-Dirac distributions with varying temperatures (see non-log. scale data with Fermi-Dirac distribution fits in the inset in **k**).

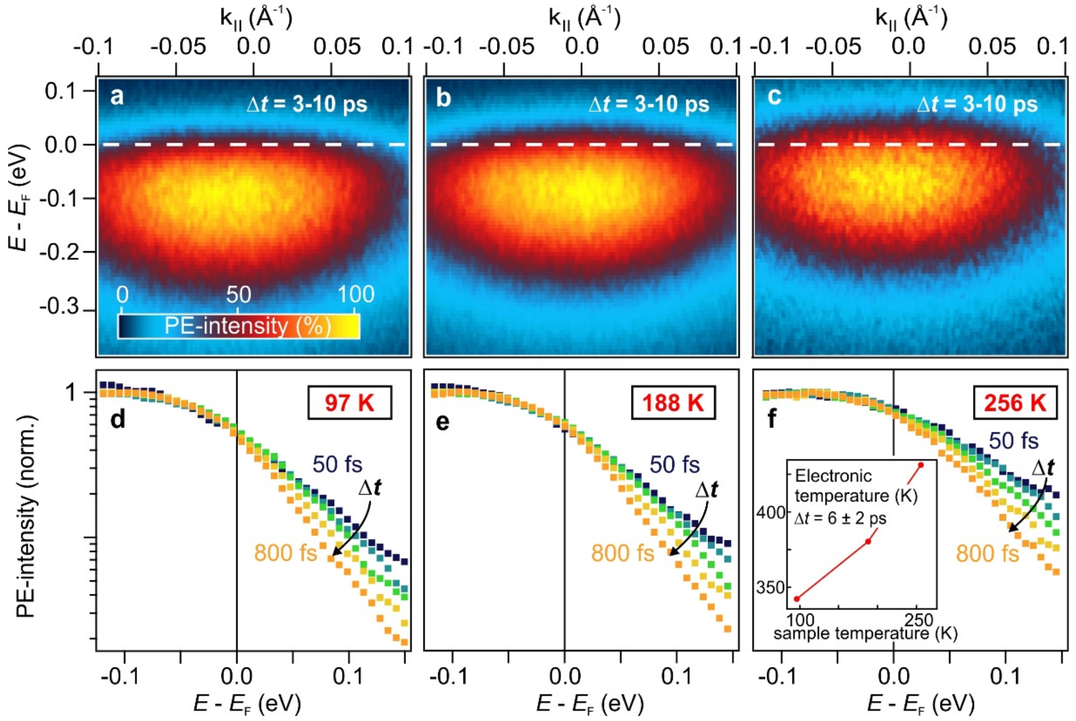

Supplementary Figure 3 Temperature dependence of the photoresponse. a-c, Angle-resolved photoinduced photoelectron intensity distribution at 97, 188 and 256 K, respectively, recorded with a pump photon energy of $h\nu_{Pump}$ = 3.43 eV and a probe photon energy of $h\nu_{Probe}$ = 6.3 eV. The pump fluence is above the threshold fluence F_C. The displayed photoelectron intensity is averaged across pump-probe delays between 3 and 10 ps. All angle-resolved spectra show a free electron-like band. **d-f**, Corresponding angle-integrated spectra exhibit the cooling of a hot electron population within the first ps, characteristic for the surface metal phase. The inset of **f** shows that, after equilibration with the lattice at a late pump-probe delay of 6 ± 2 ps, the electron (lattice) temperature also increases with the sample temperature (analysis as described in Methods). This shows that the photoinduced SMT works at least up to 256 K.
