# Supplementary material for: Ultrafast generation and decay of a surface metal
Source: Nat Commun. 2021 Feb 12;12:978. doi: 10.1038/s41467-021-21203-6 (PMC7881126; doi:10.1038/s41467-021-21203-6)
Supplement: Supplementary file 3 — Description of Additional Supplementary Files [file 41467_2021_21203_MOESM3_ESM.pdf]

Description of Additional Supplementary Files

Supplementary Movie 1

Movie of the ultrafast rise and decay of the surface metal phase
